# Assessment of eight HPV vaccination programs implemented in lowest income countries

**DOI:** 10.1186/1471-2458-12-370

**Published:** 2012-05-23

**Authors:** Joël Ladner, Marie-Hélène Besson, Rachel Hampshire, Lisa Tapert, Mike Chirenje, Joseph Saba

**Affiliations:** 1 Rouen University Hospital, Rouen, France; 2 Axios International, 7 boulevard de la Madeleine, 75001, Paris, France; 3 Axios Healthcare Development, Cleveland, USA; 4 University of Zimbabwe, Harare, Zimbabwe; 5 Epidemiology and Public Health Department, Rouen University Hospital, Hôpital Charles Nicolle. 1, rue de Germont, 76 031, Rouen cedex, France

**Keywords:** HPV vaccination, Cervical cancerp, Low and middle-income countries, Vaccine delivery, Program evaluation

## Abstract

**Background:**

Cervix cancer, preventable, continues to be the third most common cancer in women worldwide, especially in lowest income countries. Prophylactic HPV vaccination should help to reduce the morbidity and mortality associated with cervical cancer. The purpose of the study was to describe the results of and key concerns in eight HPV vaccination programs conducted in seven lowest income countries through the Gardasil Access Program (GAP).

**Methods:**

The GAP provides free HPV vaccine to organizations and institutions in lowest income countries. The HPV vaccination programs were entirely developed, implemented and managed by local institutions. Institutions submitted application forms with institution characteristics, target population, communication delivery strategies. After completion of the vaccination campaign (3 doses), institutions provided a final project report with data on doses administered and vaccination models. Two indicators were calculated, the program vaccination coverage and adherence. Qualitative data were also collected in the following areas: government and community involvement; communication, and sensitization; training and logistics resources, and challenges.

**Results:**

A total of eight programs were implemented in seven countries. The eight programs initially targeted a total of 87,580 girls, of which 76,983 received the full 3-dose vaccine course, with mean program vaccination coverage of 87.8%; the mean adherence between the first and third doses of vaccine was 90.9%. Three programs used school-based delivery models, 2 used health facility-based models, and 3 used mixed models that included schools and health facilities. Models that included school-based vaccination were most effective at reaching girls aged 9-13 years. Mixed models comprising school and health facility-based vaccination had better overall performance compared with models using just one of the methods. Increased rates of program coverage and adherence were positively correlated with the number of vaccination sites. Qualitative key insights from the school models showed a high level of coordination and logistics to facilitate vaccination administration, a lower risk of girls being lost to follow-up and vaccinations conducted within the academic year limit the number of girls lost to follow-up.

**Conclusion:**

Mixed models that incorporate both schools and health facilities appear to be the most effective at delivering HPV vaccine. This study provides lessons for development of public health programs and policies as countries go forward in national decision-making for HPV vaccination.

## Background

As of 2008, cervical cancer is the third most common cancer in women, and the seventh most common cancer overall. It is estimated that 530,000 new cases of cervical cancer were diagnosed in 2008, of which 85% occurred in less developed countries. In these countries, cervical cancer accounts for 13% of all female cancers. There were 275,000 deaths from cervical cancer in 2008 and, consistent with the incidence data, 88% of deaths occurred in developing countries [1]. Due to the relatively young age of women who develop cervical cancer, the disease is the single biggest cause of age-weighted years of life lost in the developing world [2].

Human papillomavirus (HPV) is found in 99.7% of cervical cancers worldwide, and HPV infection is considered a necessary cause of the disease [3]. Although multiple HPV types are associated with increased risk of developing high-grade cervical lesions that are precursors to cervical cancer, HPV-16 and HPV-18 are associated with almost 70% of such lesions [4]. Consequently, prophylactic vaccination against HPV, particularly types 16 and 18, reduce the incidence of cervical cancer by reducing the incidence of HPV infection. Two prophylactic HPV vaccines have been available since 2006 and have high efficacy (>90%) for preventing high-grade cervical lesions associated with HPV-16 and HPV-18 [5-9]. An analysis of cervical screening and immunization practices in Latin America and the Caribbean suggests that implementation of a universal HPV vaccination program in the context of a revised cervical cancer screening policy is the best prospect for reducing the incidence of cervical cancer in the region [10].

Despite the efficacy and availability of prophylactic HPV vaccines, a number of barriers have limited their uptake in low-resource settings, including: cost; difficulty in effectively reaching HPV vaccine target populations; competition for immunization resources as vaccines against other diseases are also being introduced; cultural issues related to the fact that HPV infection is a sexually transmitted disease; limited awareness of cervical cancer and its relationship to HPV infection; concerns about HPV vaccination with respect to safety and future fertility; negative experiences with previous vaccinations for other diseases; and political factors [11-18]. Another key challenge for implementing national HPV vaccine programs in the developing world is determining which facilities and personnel will deliver the vaccine [19,20].

In an effort to address these challenges and identify effective processes for implementing HPV vaccination among adolescent girls in low-resource settings, Axios Healthcare Development is managing the Gardasil Access Program (GAP) and is the recipient of the Gardasil donation following a pledge by Merck & Co., Inc to donate at least 3 million doses of Gardasil® [Human Papillomavirus Quadrivalent (Types 6, 11, 16 and 18) Vaccine, Recombinant]. Projects supported by the GAP are developed and implemented by local, in-country organizations or institutions. While participating programs receive a donation of HPV vaccine doses through GAP, they are otherwise supported entirely by their respective countries. A key component of the GAP is to capture the experiences and lessons learned by participating programs and institutions, and to leverage this knowledge to improve HPV vaccine access and child/adolescent immunization models. The success of each program and its ability to address the challenges listed above were assessed in order to identify trends, similarities, and differences among various strategies, and to leverage this knowledge to inform future HPV vaccination efforts.

The aim of this study was to describe the results of and key concerns in eight HPV vaccination programs conducted in seven lowest income countries through the Gardasil Access Program (GAP).

## Methods

### Setting

The GAP provides free vaccine to organizations and institutions in eligible lowest income countries, including the 3 doses of the vaccine required for a complete vaccination series. While participating projects receive free vaccine, they are then responsible for covering costs related to the importation, transportation, storage and distribution of vaccine, as well as costs for community outreach, efforts to promote the program, management of the project and data collection. The GAP enables organizations and institutions in eligible lowest income countries to gain operational experience designing and implementing HPV vaccination programs. The GAP also encourages applicants to follow World Health Organization (WHO) recommendations and guidelines for HPV vaccination, with respect to both target populations and safety vaccine administration protocols particularly in the vaccination of girls within the 9-13 years age range [21]. It should be noted that three programs were approved prior to the issuance of the WHO guidelines recommending HPV vaccination for girls aged 9-13 years.

### Inclusion procedures

Each institution interested in participating in the GAP submitted a completed application form, which collected standardized information about the primary institution, including institution type, core competencies, and catchment area; collaborating organizations; vaccine program activities (vaccination experience, vaccination procedures); training in vaccine administration and injection safety procedures; services available to store, administer, and dispose of vaccination materials; delivery model (obtaining parental/caregiver consent, type of delivery model, vaccination schedule); operational activities (communications and sensitization of key stakeholders), requested vaccine (number of vaccination sites, number of expected participants and target population).

Independent experts evaluated the submitted application using a standardized review form and made recommendations on acceptance or rejection of demand. The GAP Advisory Board then undertook a final review of each application and provided recommendations for final approval.

### Project follow-up

Participating institutions are required to submit a final project report after completion of the vaccination campaign (3 doses). Data included in this final report were: disposition of vaccine doses; number of girls receiving each dose of Gardasil, stratified inside or outside the 9-13 year age range; target audiences, key messages, methods of communication, and reactions and lessons learned with respect to communicating with stakeholder groups; cold chain logistics and distribution results; and program costs. Data on vaccine delivery models and point of vaccination were also submitted.

The programs used three models for vaccine delivery: school-based models administered vaccine at local school facilities; health facility-based models administered vaccine at health facilities, hospitals and mobile clinics; and mixed models used both schools and clinics to deliver vaccine.

### Vaccination indicators

The effectiveness of each program was determined based on two indicators: program vaccination coverage and vaccination adherence. Estimated program vaccination coverage was defined as the number of girls receiving all 3 doses of vaccine divided by the number of girls originally targeted by the project. Each program used different factors to determine the target population. Programs using health facility-based or mixed models utilized population data for the geographic area included in the campaign. Information on the number of inhabitants and number of girls included in the vaccination age range living in a specific geographic area was obtained used to calculate the target population for the vaccination program. With this methodology, the target population calculation is directly impacted by errors or inaccuracies in the census data. Programs using school-based models based their target population calculation on the number and age of girls registered at participating schools. Vaccination adherence was defined as the number of girls receiving all 3 doses of Gardasil divided by the number of girls who received a dose of vaccine. Adherence measures were calculated between dose (D)2 and D1, between D3 and D2 and between D3 and D1. All programs adhered to the standard Gardasil vaccine schedule.

For the qualitative analysis, textual data were extracted from application forms and progress reports and then indexed. This enabled the generation of specific analytical categories: government and community involvement; media, communication, and sensitization; training and human resources; challenges; and 3 models defined. Original texts were used to illustrate the results of the qualitative analysis [22].

### Qualitative analysis

Qualitative data on a variety of indicators were systematically collected and analyzed for each program in the form of field notes and transcripts. These indicators were: government and community involvement (discussions with parents and their girls; financial contribution) media, communication, and sensitization (HPV vaccination program and cervical cancer prevention); training and logistic resources; and challenges in executing the program as designed. The analysis was conducted according to the three defined delivery models. The qualitative data were preserved in textual form and then indexed to generate analytical categories. Two separate analyses were performed independently, and the results were merged and evaluated in order to generate themes that captured the range of experiences and views reported.

Three criteria were used to assess community involvement: participation of the community in the diffusion of information about the vaccination campaign; active role of community in the announcement of the three vaccination sessions; and participation of the community in the follow up of girls (reducing the number of participants lost to follow-up). Community involvement was qualified as “strong” if the three criteria were present, “medium” if two criteria were met, and “low” if only one criterion was satisfied.

### Statistical analysis

The Mann–Whitney test was used when appropriate. Comparisons between vaccination program coverage and adherence to vaccination between first and third dose and the three vaccination delivery models were tested by Kruskal-Wallis test. Correlation between the number of vaccination sites and the program vaccination coverage was tested using the Spearman correlation rank (r_s_). For all analyses, a significance level of 0.05 was used. Statistical analyses were carried out in Statview® 5.0 SAS (SAS Institute Inc.).

## Results

A total of eight programs implemented in seven countries were included: Bhutan, Bolivia, Cambodia, Cameroon, Haiti, Lesotho, and Nepal. The baseline characteristics of these programs are presented in Table 1. Projects in Bhutan, Bolivia (1), Cambodia, Haiti and Lesotho were initiated in 2009; projects in Bolivia (2), Cameroon, and Nepal were initiated in 2010.

**Table 1:** Characteristics of the 8 HPV vaccinations project included in the Gardasil Access Program, 2009-2011

No	Country	Type of responsible institution	Vaccination areas	Vaccination delivery model	Girls age group	Number of vaccination sites
1	Bhutan	MoH	Mixed	School	9-13 years*	9
2	Bolivia	NGO	Mixed	School	9-13 years	57
3	Bolivia	NGO	Mixed	Mixed	9-13 years	258
4	Cambodia	MoH	Urban	Health facility	11-18 years	1
5	Cameroon	NGO	Mixed	Health facility	9-18 years	20
6	Haiti	NGO	Rural	School	9-13 years	7
7	Lesotho	MoH	Mixed	Mixed	10-18 years	47
8	Nepal	NGO	Mixed	Mixed	9-13 years	24

*NGO*: Non-Governmental Organization.

*MoH*: Ministry of Health.

* Bhutan: age repartition: data not available.

Table 2 summarizes by program the vaccination delivery model, number of vaccination sites, the number of girls targeted for vaccination, the number of girls receiving the full vaccine course, the percentage of girls completing the vaccine course who were 9-13 years of age, estimated program coverage and adherence rates. The eight programs initially targeted a total of 87,580 girls (range [R] = 1,600–40,100), of which 76,983 (R = 1,033–33,818) received the full 3-dose vaccine course. This corresponds to an overall estimated program coverage rate of 87.8% (R = 64.5%-107.4%). Projects with coverage > 100% delivered 3 doses of vaccine to a greater number of girls than originally targeted. Between D3 and D1, the mean program adherence was 90.9% (R = 75.8%-98.7%). The number of vaccination sites was positively correlated to the program vaccination coverage (r_s_ = 0.46, p = 0.29).

**Table 2:** Number of HPV-targeted and vaccinated girls and vaccination adherence in 8 HPV-Programs, Gardasil Access Program, 2009-2011

No	Country	Vaccination delivery model	Number of vaccination sites	Number of girls targeted	Number of girls received full vaccine course	Number of girls aged 9-13 years received full vaccine course (%)	Estimated Program coverage %	Adherence to vaccination %		
								**Between**	**Between**	**Between**
								**D2-D1**	**D3-D2**	**D3-D1**
1	Bhutan	School	9	3,200	2,721	2,721 (100)*	85.0	96.8	91.3	88.3
2	Bolivia	School	57	3,480	3,739	3,739 (100)	107.4	99.8	96.6	96.1
3	Bolivia	Mixed	258	30,900	27,597	27,597 (100)	89.3	99.5	98.0	96.2
4	Cambodia	Health facility	1	2,000	2,027	186 (9.2)	101.3	98.2	97.0	95.3
5	Cameroon	Health facility	20	1,600	1,033	629 (60.9)	64.5	93.5	89.1	83.3
6	Haïti	School	7	3,300	2,884	2,884 (100)	87.4	86.7	87.3	75.8
7	Lesotho	Mixed	47	40,100	33,818	24,148 (71.4)	84.3	94.9	98.5	93.4
8	Nepal	Mixed	24	3,000	3,164	3,164 (100)	105.5	99.7	98.9	98.7
	Total	-	423	87,580	76, 983	65,068 (80.4)	87.8	96.1	94.5	90.9

* Bhutan: age repartition: data not available.

Program vaccination coverage and adherence were also stratified for the three vaccination delivery models (Table 3). The health facility model had a lower rate of coverage (77.1%) than programs using a mixed model (93.8%) or a school model (93.0%) (p = 0.74). With respect to program adherence between D3 and D1, the mixed model was most effective (96.6%), intermediate for the school model (88.6%) and the health facility model was least effective (79.7%) (p = 0.39). The estimated coverage was 94.9% for the 5 programs that vaccinated girls in the range of 9-13 years, and 80% for the three programs that vaccinated outside that age range (p = 0.25). Program adherence was 91.0% for the projects within the 9-13 years age range, and 84.3% for programs vaccinating outside the range (p = 0.49).

**Table 3:** Number of HPV-vaccinated girls and vaccination adherence according to the vaccination delivery model in 8 the HPV-Programs, Gardasil Access Program, 2009-2011

Vaccination delivery model	Number of Programs	Number of girls received full vaccine course	Estimated Program coverage %	Adherence to vaccination %		
				**Between**	**Between**	**Between**
				**D2-D1**	**D3-D2**	**D3-D1**
School	3	9,344	93.0	94.3	91.7	88.6
Health facility	2	2,742	77.1	92.4	85.5	79.7
Mixed	3	64,897	93.8	98.0	97.3	96.6

A variety of communication vehicles were used, including community meetings informational posters, flyers, television, radio, and newspapers. Many programs mentioned the need to include basic information on cervical cancer in very plain language, and this was particularly challenging in Bolivia and Lesotho, in which local language equivalents for “cervix” do not exist.

Community involvement was strong in five of the seven programs for which data were available (data were not available for the project conducted in Bhutan), medium in one project and low in one project. Strong community involvement was reported for the definition of key messages, recruitment of participants and follow-up with participants. The community was involved in the announcement of the vaccination sessions in less than 30% of the projects.

Qualitative results from the Haiti project suggest that establishing formal involvement of community members in all aspects of the project ensured community ownership. In Cambodia and Cameroon, a voluntary small financial contribution to cover the administrative costs of participation ensured that communities and families perceived an intrinsic value in the vaccine and vaccination services while also helping support a minimal part the costs of the program. Experiences in the Cameroon project also suggest that more in-depth discussion sessions with parents and caregivers, and evaluation of the knowledge of and attitudes toward HPV vaccination in these audiences will be important to the success of future HPV vaccination campaigns. Establishing a status symbol type reward for girls who complete the full vaccine course, such as bracelet (Nepal) or T-shirt (Haiti) helped to create demand for the vaccine among the girls themselves.

Qualitative analysis of the eight programs identified several trends and common issues. Key insights from the school models were: (i) a high level of coordination and logistics are needed to facilitate vaccination administration; (ii) there is a lower risk of girls being lost to follow-up; (iii) loss to follow-up was often associated with transfer of students to new schools, family migration, holidays and school calendar conflicts; (iv) vaccinations should be conducted within the academic year to limit the number of girls lost to follow-up.

Key benefits identified with clinic models were: (i) immediate adverse events reports are exhaustively collected in the clinic setting; (ii) the routine presence of human and other resources needed to administer vaccine facilitated administration of vaccine outside of the planned vaccination schedule; (iii) clinic sites had vaccine storage facilities readily available onsite.

The mixed models exhibited key points from both the school and clinic models. In the mixed models, the availability of clinic resources helped to ensure vaccination of those girls who missed their vaccination day at school. The increase in the number of vaccine distribution sites required the continual evaluation of cold chain capacity at schools and on going monitoring of the distribution and stock of vaccine across various sites.

## Discussion

Overall, the eight programs included in this study performed very well by the two indicators used. Mean program coverage across all programs was 88% and mean adherence between D3 and D1 was 91%. The highest program vaccination coverage was reported in the Bolivia 1 program. Such a high coverage rate in a school-based model was somewhat unexpected. However, it should be noted that there was a high demand for HPV vaccination from other nearby schools not originally included in the program. The program manager and health authorities extended the vaccination period for an additional two weeks in order to accommodate this demand. Due to the extended time frame and the inclusion of girls from schools not included in the initial targeting plan, the program was able to vaccinate more girls than originally expected, which accounted for a coverage rate greater than 100%.

School-based vaccination delivery methods were most effective at reaching girls within the WHO-recommended age range, which is likely due to the fact that girls aged 9-13 years are not usual “clients” of the health care system. Additionally, few health systems have robust follow-up systems in place for ensuring that participants visit the clinic for each of the three scheduled vaccinations. It should also be noted that the Cambodia project, which used a health facility-based model, was approved prior to the issuance of the WHO age recommendations for HPV vaccination and included girls older than 13 years of age. This certainly contributed to the lower percentage of girls within the age range for health facility-based models compared with the other models.

However, school-based projects faced several challenges when vaccination dates occurred outside of scheduled school days. It was exponentially more difficult to prevent loss to follow-up in these instances and in some cases, clinic and/or door-to-door follow up was necessary to complete the 3 doses.

Interestingly, there was a trend toward increased rates of project coverage and project adherence with an increasing number of vaccine administration sites. This trend makes sense given that more sites increase the capacity for vaccine administration and, in fact, vaccine utilization is higher when administration sites are easily accessible [20]. However, it was not necessarily a given that small pilot programs such as those described here would have the resources or expertise to successfully manage multiple sites. These results suggest that local organizations can successfully implement relatively larger HPV vaccination campaigns and that additional sites should be considered in order to increase access to and delivery of HPV vaccines.

Mixed models yielded better overall performance (program vaccination coverage and adherence indicators) compared with school or clinic models. This may be due to promotion of the program through health and educational channels. Community participation appeared to be a less effective recruitment driver than channels that utilized schools and clinics. The ability to deliver vaccination at school or a clinic site increased the likelihood that participants would receive their vaccinations on time even in cases of school relocation, family migration or vaccination schedules that coincided with school holidays. Adherence in school-based models may also have been negatively affected by the 2010 earthquake in Haiti, which occurred between D2 and D3 of the school-based Haiti program.

The feasibility and success of a similar mixed model has also recently been demonstrated in Peru [23]. The data show that inclusion of HPV vaccination did not diminish health facilities’ capacity to administer routine infant vaccination programs. Additionally, an analysis of HPV vaccine acceptability in Botswana found that public or community clinics would be the most common place at which people would get HPV vaccine and that 74% of study participants indicated that they would have their daughters vaccinated against HPV at school if the vaccine was available there[16]. These findings further support the utility of a mixed model encompassing both school and clinic-based vaccine delivery.

Based on the success and challenges of these eight GAP programs, it is recommended that future programs should conform to local norms for other vaccinations with respect to signed consent by girls and their families who chose to receive HPV vaccination. Additionally, specific resources should be allocated to sensitize and train schoolteachers to assist in recruitment of and follow-up with girls during HPV vaccination campaigns. Such training was deemed an essential component to the success of a school-based HPV vaccine program in Peru [23]. Teachers and headmasters were also considered decision-makers with respect to school-based HPV vaccination in Peru, Uganda and Vietnam, and schools were identified as effective sources for community health education programs about cervical cancer [11,12]. Effective use of schools as venues for HPV vaccine programs has also been identified as an important factor in the successful adoption of HPV vaccine in low-resource settings [19].

The high levels of estimated coverage and vaccination adherence achieved with these programs could be due to their relatively small size and close monitoring, and the fact that resources have been allocated to them specifically to conduct HPV vaccination activities. For the current design of the GAP, the analyses of these projects are a measure of the ability of a project or institution to recruit participants and ensure that they receive all 3-vaccine doses. These analyses do not assess the ability of a project or institution to provide HPV vaccine coverage to an entire population. Consequently, the two indicators used to assess programs undertaken are distinct from those related to measuring vaccine coverage.

In addition to a small sample size of only eight programs, this study has several limitations. The use of census data to determine the number of eligible girls within the geographic regions of health facility-based or mixed model programs is an imprecise methodology that could give rise to errors in determining target population. Given that target population is the denominator in the calculation used to determine program vaccine coverage, error in determining the number of eligible girls who could participate in a program will impact coverage rates. This potential for error is somewhat less of an issue in the three programs that used school-based models because school enrolment data is more precise than census data. Although population density could also impact program vaccination coverage, data on population density were not systematically collected and were thus not included in the analysis reported here.

Three programs had program coverage greater than 100%: Bolivia 1 (school-based model), Cambodia (health-facility model), and Nepal (mixed model). It is possible that errors in the census data used to calculate the denominator of the coverage estimation account for these results in the Cambodia and Nepal programs. However, it is also possible that demand for HPV vaccination near to but not within the original coverage area resulted in the recruitment of more girls than expected. Information campaigns and community involvement may have been more effective than expected in some areas or programs, which also could have increased recruitment above original estimates. However, it is also possible that demand for HPV vaccination near to but not within the original coverage area resulted in the recruitment of more girls than expected. Information campaigns and community involvement may have been more effective than expected in some areas or programs, which also could have increased recruitment above original estimates.

Cost analysis data and model projections on cost-effectiveness of different HPV vaccination programs in low- and middle-income countries are not included because the cost data collected were incomplete. Additionally, the projects reported on here are pilots developed at the community level, and may not be applicable to larger regional or national campaigns that may comprise diverse socio-economic, religious, and cultural constituencies. Optional preventive strategies, regional strategies, or mixed strategies in rural low-income regions could play an important role in addressing the challenges associated with HPV vaccine coverage, access, and acceptance [24]. Data from the projects described here may enable diverse approaches that allow individual countries or regions to maximize the coverage of HPV vaccination campaigns.

## Conclusions

These results suggest that local organizations and institutions can implement successful HPV vaccination campaigns. Mixed models comprising both school and health facility settings appear to be the most effective at delivering HPV vaccine to target girls aged 9-13 years. Such mixed models may increase the performance of other vaccination programs and may also be relevant to the delivery of other care or preventive services for school-aged children. Partnership between schools and health facilities may also increase the delivery of services to girls who are not in school, although this may require intensive resources in order to reach those who do not have formal or routine contact with schools or health programs. The data reported here provide lessons for development of public health programs and policies as countries go forward in national decision-making for HPV vaccination.

## Competing interests

The authors declare no competing interests.

## Authors’ contribution

JL conceived the study, wrote the manuscript and performed the analysis. MHB conceived the study, collected data and wrote the manuscript. RH participated in the design study and helped to draft the manuscript. LT and MC helped to draft the manuscript. JS conceived the study and helped to draft the manuscript. All authors read and approved the final manuscript.

## Pre-publication history

The pre-publication history for this paper can be accessed here:

http://www.biomedcentral.com/1471-2458/12/370/prepub
